# Psychometric properties of the Urdu version of the EORTC QLQ-H&N35 (European organization for research and treatment of cancer head and neck module) quality of life tool

**DOI:** 10.1186/s40359-022-00900-x

**Published:** 2022-08-07

**Authors:** Nida Zahid, Russell Seth Martins, Wajeeha Zahid, Iqbal Azam, Mubasher Ikram, Aneesa Hassan, Shireen Shehzad Bhamani, Adnan Abdul Jabbar, Nargis Asad, Shabbir Akhtar, Moghira Iqbaluddin Siddiqui, Mohammad Sohail Awan, Khabir Ahmad

**Affiliations:** 1 Department of Surgery, Aga Khan University Hospital, Karachi, Pakistan; 2 Medical College, Aga Khan University Hospital, Stadium Road, P.O. Box 3500, Karachi, 74800 Pakistan; 3 Department of Community Health Sciences, Aga Khan University Hospital, Karachi, Pakistan; 4 School of Nursing and Midwifery, Aga Khan University Hospital, Karachi, Pakistan; 5 Department of Oncology, Aga Khan University Hospital, Karachi, Pakistan; 6 Department of Psychiatry, Aga Khan University Hospital, Karachi, Pakistan

**Keywords:** Oral Cancer, Quality of life, Validity, Reliability, Correlation, Urdu, Anxiety, Depression, Resilience

## Abstract

**Background:**

We translated and validated the Urdu version of the European Organization for Research and Treatment of Cancer Quality of Life (QoL) Questionnaire’s Head and Neck (H&N) Cancer Module (EORTC QLQ-H&N35) and assessed its convergent and discriminant validity by examining correlations of QoL with depression, anxiety, and resilience.

**Methods:**

We translated the EORTC QLQ-H&N35 according to EORTC instructions. Patients at a tertiary care hospital in Pakistan completed a survey consisting of Urdu versions of EORTC QLQ-C30 (core QoL tool), QLQ-H&N35, Hospital Anxiety and Depression Scale, and Wagnild and Young Resilience Scale (RS-14). Content validity, convergent validity, discriminant validity, and reliability (using Cronbach’s alpha) of the EORTC QLQ-H&N35 were assessed.

**Results:**

Our sample comprised 250 patients with H&N cancer, most commonly oral (82%). The Urdu translations were comprehensible for all patients. The Cronbach alpha for QLQ-H&N35 multi-item domains ranged from 0.75 to 0.98 (acceptable to excellent), barring “Senses Problems”, which was less than the generally acceptable level (0.50). The patient-reported content validity index (CVI) scores for relevance and clarity of the Urdu version of the QLQ-H&N35 were 0.93 and 0.92, respectively (both excellent). Our results revealed weak bidirectional correlations of the QLQ-H&N35 with resilience, depression, and anxiety, showing good discriminant validity. A weak-to-moderate but significant negative correlation (r: − 0.185 to − 0.613; *p* < 0.01) was seen between the QLQ-H&N35 and the global QoL measure of the QLQ-30.

**Conclusion:**

Our Urdu translation of the EORTC QLQ-H&N35 demonstrated validity comparable to previous studies, with good discriminant construct validity when measured against resilience, depression, and anxiety. An issue of concern is the poor internal consistency of the “Senses Problems” domain. Nevertheless, the Urdu translation produced in this study serves as a valid and reliable measure to measure QoL in H&N cancer in clinical or research settings in Pakistan.

**Supplementary Information:**

The online version contains supplementary material available at 10.1186/s40359-022-00900-x.

## Background

Head and neck (H&N) cancers are the sixth most common cancer worldwide [1], and are responsible for more than 400,000 deaths annually [2]. Around 40% of the global H&N cancer burden arises in South Asia [3], where the incidence rate exceeds 20/100,000, with oral cancers being the commonest subtype in this region [2]. In Pakistan, a lower-middle-income country (LMIC) in South Asia, H&N cancers represent > 20% of cancers in males and > 10% of cancers in females [4]. In comparison to most other types of malignancies, H&N cancers are associated with debilitating levels of distress that culminate in depression, anxiety, and even suicide [5].

Health-related quality of life (QoL) is an increasingly important consideration in cancer management [6], and encompasses patients’ physical, emotional, and psychosocial well-being and functionality [7]. The critical levels of distress experienced by patients with H&N cancer stem from the physical symptom burden, losses of functionality, and social stigma associated with the disease and its management [8]. Between 22 and 57% of patients with H&N cancer develop symptoms of depression (such as persistently low mood and anhedonia) and/or anxiety (such as excessive worry), with mental health being strongly associated with worse QoL amongst patients with H&N cancer [9]. However, resilience, which is the ability of individuals to remain physically, emotionally and cognitively functional during periods of distress, helps protect against depression and anxiety [10], and improves QoL [11], amongst patients with H&N cancers. A theoretical framework published by Zahid et al. in 2019 hypothesized resilience to be a function of external stressors, individuals’ coping abilities, and family and social support, with resilience positively influencing QoL [12]. With regards to anxiety and depression, it is important to understand that these mental health issues share a two-way reciprocal relationship with QoL, with poor QoL being both a predictor and outcome of anxiety and depression [13, 14].

Measuring QoL in patients with H&N cancer is important for its routine integration into clinical management and goal-setting, as well as its inclusion as an outcome in clinical trials. Though several tools exist to measure the QoL of patients with H&N cancer, the EORTC QLQ-C30 (European Organization for Research and Treatment of Cancer Quality of Life Questionnaire) and its head and neck cancer-specific module QLQ-H&N35 (EORTC QLQ-Head & Neck35) are popular, reliable, and valid measures [15, 16]. The EORTC tools cover a broad range of physical and psychological symptoms, as well as the impact on daily functioning, and their psychometric properties remain consistent across translations and cultures.

Global differences in languages and cultures have resulted in extensive translations of the EORTC QLQ-C30 and QLQ-H&N35 into many different languages. The EORTC QLQ-C30 has been used in over 3000 studies to date and has been translated and approved in over 100 languages [17], including Urdu [18, 19]. The EORTC QLQ-H&N35 has been less widely translated and validated, and never before in a Pakistani population in the Urdu language. Anxiety and depression, measured by tools such as the Hospital Anxiety and Depression Scale (HADS) [20] and others [21], have been used to explore the discriminant validity of the EORTC QLQ-H&N35. These mental health outcomes have shown weak-to-moderate correlation with QoL as measured by the QLQ-H&N35. Additionally, though resilience has not previously been employed in the validation process for the QLQ-H&N35, it has been used successfully while validating the EORTC QLQ-30 [19]. Moreover, resilience has been found to have a strong, positive, significant association with QoL as measured by the QLQ-H&N35, which further justifies its use in the validation process of the same [11, 19, 22].

There is an acute need to use proven measures such as the EORTC QLQ-C30 and QLQ-H&N35 to explore the QoL of patients with H&N cancer in Pakistan. Owing to the high prevalence of illiteracy in Pakistan, the original English tools have limited applicability. This prevents the measurement of QoL amongst H&N cancer patients in Pakistan, which precludes the inclusion of QoL as a management outcome. Urdu is spoken and understood across most cultural and ethnic groups is Pakistan and emerges as the language of choice for the translation of a QoL measure for general use in the country. Thus, this study aimed to translate the EORTC QLQ-H&N35 into Urdu and validate this translation amongst patients with H&N cancer in Pakistan. Moreover, since depression, anxiety, and resilience have previously been used successfully to explore the construct and discriminant validity of QoL [19–21], we also aimed to explore their correlation with QoL to support our validation of the EORTC QLQ-H&N35. Overall, we aimed to answer the following question: Does an appropriately translated Urdu version of the EORTC QLQ-H&N35 provide a sufficiently valid measure of QoL in patients with H&N cancer.

## Methods

### Study design and setting

This validation study was conducted as a survey at the Aga Khan University Hospital (AKUH), a Joint Commission International Accreditation (JCIA-accredited) hospital in Karachi, Pakistan, between November 2019 and May 2020. The ethics review committee of AKUH approved the protocol for this study (Reference Number: 5154-Sur-ERC-17), which has subsequently been published by the authors [12].

### Study subjects

Our population of interest was all adult (≥ 18 years) patients treated for H&N cancer at AKUH. Inclusion criteria included currently being ≥ 4 weeks post-initiation of treatment, and having resided in Pakistan for at least the past 3 months. The latter criterion was included to ensure validation of the EORTC QLQ-C30 and QLQ-H&N35 tool in the sociocultural context and setting of Pakistan, as patients residing in Pakistan may experience QoL differently to their counterparts residing in other countries.

Patients were excluded if they had a history of psychiatric illness or were on psychiatric medications, or had serious comorbid conditions such as stroke or renal failure. Patients with hypertension (HTN), chronic obstructive pulmonary disease (COPD), or type 2 diabetes mellitus (T2DM) were not excluded from the sample, due to their high prevalence amongst Pakistani patients [23].

### Sample size calculation and participant recruitment

A minimum sample size of 250 was calculated using the one population mean formula, a standard deviation (SD) of 20, 5% level of significance with precision of 2.5, and by adjusting the sample size for 10% rate of incomplete responses [12].

Participants were recruited via non-probability consecutive sampling. Research assistants approached patients with H&N cancer who were visiting AKUH for scheduled appointments. After screening patients for eligibility and obtaining their informed consent, the research assistants administered the study survey to the patients. Though the EORTC tools are generally self-administered, we decided to conduct interviews so as to ensure the inclusion of patients who lacked the literacy to read. This was similar to as was done in the validation of the Moroccan Arabic version of the EORTC QLQ-H&N35 [24].

### Study measures

The primary tool validated in this study was the European Organization for Research and Treatment of Cancer Head & Neck Module (EORTC QLQ-H&N35), which is co-administered with the EORTC QLQ-C30 core QoL in cancer questionnaire. The authors obtained permission from the EORTC for the use, translation, and validation of both tools. Furthermore, to assess the correlation of QoL with depression, anxiety, and resilience, the Wagnild and Young’s Resilience Scale (RS-14) and Hospital Anxiety and Depression Scale (HADS) were also used in this study. The final instrument thus comprised of a preliminary section on sociodemographic and clinical variables, followed by the EORTC QLQ-C30, EORTC QLQ-H&N35, HADS and RS-14:

- *EORTC QLQ-C30*: The EORTC defines QoL as “the subjective perceptions of the positive and negative aspects of cancer patients’ symptoms, including physical, emotional, social, and cognitive functions and, importantly, disease symptoms and side effects of treatment.” The EORTC QLQ-C30 has previously been translated to Urdu and validated [19] by the authors of the current study, and the same Urdu translation was used in this study. As a summary, the Cronbach’s alpha for the Urdu version is 0.86, while the content validity index (CVI) scores for clarity and relevance are 0.98 and 0.96, respectively [19]. We also recalculated the Cronbach’s alpha for the current sample of H&N35, and found it to have a value of 0.84. This 30-item QoL measure comprises of a global health and QoL scale, five multi-item functional domains (cognitive, physical, role, emotional and social), three symptom domains (pain, fatigue, and nausea and vomiting), single item measures for other commonly experienced symptoms (including sleep disturbance, constipation, diarrhea, appetite loss, and dyspnea), and perceived economic impact of the cancer disease and management [15]. A 4-point Likert scale (1: ‘not at all’; to 4: ‘very much’) is used to score all items, barring two in the global health/QoL scale which instead use 7-point linear analog scales [16]. For the functional and global QoL items, higher scores imply better functionality and QoL (favorable conditions). For symptom domains, higher scores entail greater symptomatology (unfavorable conditions).
- *EORTC QLQ-H&N35*: A 35-item QoL measure specifically for patients with head and neck neoplasms [16]. The H&N35 comprises seven multi-item scales assessing pain, swallowing, senses (taste and smell), speech, social eating, social contact, and sexuality. In addition, eleven single items are also included. Items 1–30 are scored on 4-point Likert scales. Items 31–35 have a ‘‘no/yes’’ response format. The scores are transformed into a 0–100 scale, with a higher score indicating more unfavorable conditions for all items [25].
- *RS-14*: As measured by the RS-14, resilience is defined as “the capacity to live with purpose, perseverance, equanimity, authenticity, and self-reliance, in the face of adversity”. This 14-item tool measures five core features of resilience (perseverance, purposeful life, self-reliance, equanimity and existential loneliness), and uses a 7-point Likert Scale to compute a total score [26]. A higher total score indicates greater resilience. The Urdu version of RS-14 was employed, which is validated with a good Cronbach’s alpha of 0.763 [27]. We re-evaluated the internal consistency of the Urdu version RS-14 and observed an excellent Cronbach’s alpha of 0.889 in the current population of patients with H&N cancer.
- *Hospital Anxiety and Depression Scale (HADS)*: This 14-item measure uses a 4-point ordinal scale to assess depression and anxiety, with a lower score indicating more favorable an outcome. The HADS characterizes anxiety as persistent feelings of panic, fear, restlessness or tension, and depression as persistent feelings of anhedonia and low mood. The Urdu translation of the HADS [28] was employed, as it has been found to have a good overall Cronbach’s alpha of 0.84 (0.64 and 0.82 for depression and anxiety subscales, respectively) [29]. In our current sample of patients with H&N cancer, the overall Cronbach’s alpha was 0.88, with those of the anxiety and depression subscales being 0.79 and 0.84, respectively.

### Translation of study measures and pilot testing

Translation of the EORTC QLQ-C30 and QLQ-H&N35 was performed according to criteria and standards delineated by the EORTC [30] and the COSMIN Study Design Checklist for Patient-reported Outcome Measure Instruments [31]. First, two translators independently translated the EORTC QLQ-C30 and QLQ-H&N35 from English into Urdu. These translators were bilingual, with complete professional proficiency in English and native-level proficiency in Urdu, and with > 7 years of experience of health-related survey translation. One of the translators was made aware of the constructs that the tools were designed to gauge, while the other translator was naïve to the measured constructs. This was so as to limit bias and pick minor discrepancies. The two Urdu versions were then consolidated to one Urdu version, which then underwent independent backwards translation to English by two other translators. Both translators had native proficiency in Urdu and full professional proficiency in English, and were naïve to the constructs measured by the tools. A single English version was consolidated from the two backwards translations, and reviewed for consistency with the original English version of the tools. Discrepancies were identified and settled with the help of a final independent translator who was aware of the intended purpose of the tools.

The difficulties encountered during the translation process are described in the (Additional file 1). Pilot testing of the preliminary Urdu translations were performed on 10% of calculated minimum required sample size, i.e., 25 patients with H&N cancer who were native Urdu speakers. These patients were asked to comment on any questions they found hard to understand, hard to answer, upsetting, confusing, or offensive. This served as linguistic validation of the comprehensibility, in accordance with EORTC guidelines [32]. In addition, patients were also required to rate the comprehensibility of each item using a Likert scale of 1–4 (as described in the subsection on Content Validity Index). No major problems were identified during the pilot testing, with only a few minor changes being made to finalize the Urdu translations of both tools. The Urdu translation of the EORTC QLQ-H&N35 is appended as (Additional file 2).

### Statistical analysis

SPSS Statistics for Windows version 23.0 was used to analyze the data (IBM Corp., Armonk, N.Y., USA). Data analysis was carried out by two members of the research team. Frequencies and percentages were used to represent categorical variables. Means and standard deviations were used to represent numerical variables. The Pearson correlation coefficients were calculated to determine the convergent validity of the condition-specific QoL measure EORCT QLQ-H&N35 with the validated core QoL tool EORCT QLQ-30, as well as the discriminant validity of QLQ-H&N35 with the RS-14 and HADS. The Cronbach's alpha coefficient was used to estimate reliability of multi-item subscales, with a value ≥ 0.70 considered acceptable. Interscale correlations were computed for the QLQ-H&N35.

The content validation of the QLQ-H&N35 tool was performed according to the criteria described in the COSMIN Study Design Checklist for Patient-reported Outcome Measure Instruments [31]. This procedure employs Lawshe’s approach to content validity [33]. Five experts (a psychologist, mental health researcher, biostatistician, epidemiologist, and otolaryngologist) were asked to rate the relevance and clarity of each item of the tool on a Likert scale of 1–4. Similarly, patients participating in the pilot testing were also required to rate the relevance and clarity of each item using a Likert scale of 1–4. These ratings were then used to calculate a content validity ratio (CVR) for each item. The critical value for the CVR calculated for an expert panel of five raters is 1, while the critical value for the panel of 25 pilot-tested patients was 0.440 [34]. The content validity index (CVI) scores for the tool’s clarity and relevance were calculated by averaging the CVR of individual items. This yields a CVI score ranging from 0 to 1 (1 = perfect agreement; 0 = no agreement). Since the EORTC QLQ-H&N35 is pre-designed and has been used in studies around the world in its current form, its comprehensiveness is well-established and was not assessed. A *p* value < 0.05 was regarded as significant for all analyses.

## Results

### Sociodemographic and clinical characteristics

Our sample consisted of 250 patients with H&N cancer. The patients’ mean age was 51.59 years, with 198/250 (79%) male. Most patients had acquired formal education (87%). Urdu was the mother tongue of about 50% patients. 87% of the patients were married and around half (53%) lived with extended families. Around two-thirds (64%) were not currently working. The most common cancers were oral (82%) and laryngeal (14%). The majority of patients had undergone a biopsy only (74.8%). Around half (52.8%) had undergone combination radiotherapy and chemotherapy. Treatment was complete for 56.8% of patients (Table 1).

**Table 1:** Participants’ Socio-demographic and Disease related characteristics

Variables	N = 250
	n (%)/ Mean ± SD
Age (years)	51.59 ± 13.23
Gender	
Male	198 (79.2)
Female	52 (20.8)
Formal schooling	
Yes	218 (87.2)
No	32 (12.8)
Marital status	
Married	218 (87.2)
Single	14 (5.6)
Other	18 (7.2)
Family structure	
Extended	134 (53.6)
Nuclear	116 (46.4)
Working Status	
Working	89 (35.6)
Not working	161 (64.4)
Spouse’s working status	**N = 218**
Working	36(16.5)
Not working	182 (83.5)
Monthly household income (PKR/USD)	
No income	18 (7.2)
PKR 2000–25,000 ($13-$151)	40 (16.0)
PKR 25,000–40,000($151-$242)	26 (10.4)
PKR 40,000–80,000($242-$484)	69 (27.6)
PKR 80,000–170,000 ($484-$1028)	97 (38.8)
Tumor type	
Oral Cancer	205 (82.0)
Laryngeal	35 (14.0)
Others	10 (4.0)
Surgical intervention	
Only biopsy	187 (74.8)
Only total resection	7 (2.8)
Multiple interventions	4 (1.6)
No surgical intervention	52 (20.8)
Adjuvant therapy	
Chemotherapy	12 (4.8)
Radiotherapy	46 (18.4)
Combination	132 (52.8)
No adjuvant therapy	60 (24.0)
Treatment stage for head and neck patients	
On-going	108 (43.2)
Complete	142 (56.8)
Feeding tube needed	104 (41.6)
Tracheostomy needed	19 (7.6)

Bolded text was to represent the subset sample size of patients with a spouse

### Internal consistency or reliability

The internal consistency of 6/7 multi-item symptom domains of QLQ- H&N35 ranged from acceptable to excellent (Cronbach’s alpha range: 0.75–0.98; *p* < 0.001), while the internal consistency of “Senses Problems” was subpar at 0.50. The greatest floor effects were seen for the “Sticky Saliva”, “Teeth”, and “Trouble with Social Contact” items, whereas the greatest ceiling effects were seen with “Weight Gain”, “Nutritional Supplements” and “Pain Killers” (Table 2).

**Table 2:** Internal consistency of QLQ-H&N35

QLQ-H&N35 symptom domains	Number of ITEMS	Cronbach’s alpha (*p* value)	Median	Mean	SD	Floor (%)^a^	Ceiling (%)^b^
Pain	4	0.75 (< 0.001)*	8.33	13.93	19.38	45.2	0.4
Swallowing	4	0.85 (< 0.001)*	8.33	14.33	17.00	45.2	1.2
Senses problems	2	0.50 (< 0.001)*	50.00	61.53	27.49	28.4	4.8
Speech problems	3	0.88 (< 0.001)*	22.22	32.31	34.24	34.0	11.2
Trouble with social eating	4	0.91 (< 0.001)*	16.67	30.77	33.53	36.4	8.8
Trouble with social contact	5	0.90 (< 0.001)*	0.00	14.88	23.00	52.4	0.8
Less sexuality	2	0.98 (< 0.001)*	33.33	44.77	37.07	28.8	18.4
Teeth	1	–	0.00	20.80	34.49	68.0	11.2
Opening mouth	1	–	50.00	46.00	38.43	32.2	21.2
Dry mouth	1	–	33.33	40.27	34.27	30.0	14.4
Sticky saliva	1	–	0.00	20.53	35.17	69.6	12.4
Coughing	1	–	0.00	24.80	30.44	50.8	6.8
Felt ill	1	–	33.33	25.87	29.23	46.0	6.0
Pain killers	1	–	100.00	70.00	45.92	30.0	70.0
Nutritional supplements	1	–	100.00	71.60	45.18	28.4	71.6
Feeding tube	1	–	0.00	49.60	50.10	50.4	49.6
Weight loss	1	–	100.00	55.60	49.79	44.4	55.6
Weight gain	1	–	100	73.20	44.38	26.8	73.2

*SD* Standard deviation

*Significant at *p*-value < 0.05 by reliability analysis

^a^Percentage of patients with highest recorded score

^b^Percentage of patients with lowest recorded score

### Content validation

The expert-reported CVI scores for relevance and clarity of the Urdu version of the EORTC QLQ-H&N35 tool were 0.88 and 0.84, respectively, indicating good agreement among the five experts. The patient-reported CVI scores for relevance and clarity of the Urdu version of the QLQ-H&N35 were 0.93 and 0.92, respectively. These results indicate excellent agreement for clarity and relevance.

### Convergent and discriminant validity

When assessing correlations between EORTC QLQ-30 and QLQ-H&N35, we observed a significant weak-to-moderate negative correlation of the 14 symptoms domains of QLQ-H&N35 with global QoL measured by the QLQ-30 (r ranging: − 0.19 to − 0.61; *p* < 0.01). We also observed significant weak-to-strong negative correlations with the functional domains of QLQ-30 (r range: − 0.09 to − 0.90; *p* < 0.05). The strongest correlation of − 0.90 was observed between role function domain (QLQ-30) and swallowing (QLQ-H&N35).

Our results also showed a weak but significant positive correlation between 4 symptoms domains of QLQ-H&N35 (Pain Killer, Nutritional Supplements; Feeding tube; and Weight Loss;) and global QoL measured by QLQ-30 (r: 0.20–0.37; *p* < 0.05) and with the functional scales of QLQ-30 (r: 0.17–0.37; *p* < 0.05). However, there was a weak but significant negative correlation between weight gain (QLQ-H&N35 and physical functioning and emotional functioning (QLQ-30) (r: − 0.14 and − 0.15; *p* < 0.05). We also observed a significant weak-to-moderate positive correlation (r ranging: 0.17–0.51; *p* < 0.05) between 15 symptom domains of QLQ-H&N35 and 9 symptom domains of QLQ-30. In addition, we observed weak-to-moderate negative correlation between 9 symptoms domain of QLQ-30 and 4 symptom domains of QLQ-H&N35 (r: − 0.14 to − 0.36; *p* < 0.05). Table 3 presents these results.

**Table 3:** Correlation of EORTC QLQ-C30 and QLQ-H&N 35 (Convergent Validity)

	P	SW	SN	SP	TSE	TSC	LS	T	OM	DM	SS	C	FI	PK	NS	FT	WL	WG
GS	− 0.43**	− 0.61**	− 0.55**	− 0.45**	− 0.52**	− 0.56**	− 0.39**	− 0.31**	− 0.35**	− 0.19**	− 0.28**	− 0.26**	− 0.59**	0.20**	0.23**	0.37**	0.25**	− 0.10
PF	− 0.17**	− 0.53**	− 0.36**	− 0.34**	− 0.29**	− 0.39**	− 0.36**	− 0.14 *	− 0.11	− 0.09	− 0.13 *	− 0.15 *	− 0.47**	0.04	0.24**	0.27**	0.27**	− 0.14 *
RF	− 0.38**	− 0.90**	− 0.46**	− 0.42**	− 0.43**	− 0.37**	− 0.34**	− 0.34**	− 0.31**	− 0.19**	− 0.37**	− 0.12	− 0.52**	0.12	0.17**	0.33**	0.27**	− 0.06
EF	− 0.42**	− 0.51**	− 0.59**	− 0.40**	− 0.45**	− 0.62**	− 0.40**	− 0.27**	− 0.28**	− 0.18**	− 0.26**	− 0.26**	− 0.60**	0.18**	0.23**	0.24**	0.31**	− 0.15 *
CF	− 0.38**	− 0.42**	− 0.37**	− 0.31**	− 0.29**	− 0.38**	− 0.37**	− 0.24**	− 0.20**	− 0.26**	− 0.19**	− 0.05	− 0.40**	0.09	0.21**	0.06	0.17**	− 0.02
SF	− 0.36**	− 0.61**	− 0.56**	− 0.55**	− 0.61**	− 0.65**	− 0.44**	− 0.35**	− 0.33**	− 0.27**	− 0.37**	− 0.24**	− 0.63**	0.12	0.24**	0.37**	0.26**	− 0.04
F	0.44**	0.38**	0.49**	0.34**	0.34**	0.45**	0.21**	0.19**	0.26**	0.13 *	0.26**	0.37**	0.51**	− 0.23**	− 0.21**	− 0.29**	− 0.28**	0.10
NV	0.43**	0.45**	0.83**	0.35**	0.40**	0.42**	0.32**	0.30**	0.29**	0.25**	0.47**	0.28**	0.45**	− 0.26**	− 0.17**	− 0.30**	− 0.31**	0.17**
P	0.50**	0.45**	0.50**	0.41**	0.40**	0.45**	0.28**	0.25**	0.26**	0.17**	0.25**	0.26**	0.62**	− 0.31**	− 0.23**	− 0.32**	− 0.31**	0.10
DY	0.27**	0.41**	0.28**	0.30**	0.29**	0.35**	0.16 *	0.09	0.17**	0.10	0.26**	0.33**	0.30**	− 0.12	− 0.08	− 0.24**	− 0.22**	0.08
I	0.22**	0.51**	0.40**	0.43**	0.49**	0.43**	0.44**	0.18**	0.21**	0.22**	0.28**	0.09	0.52**	− 0.14*	− 0.36**	− 0.27**	− 0.29**	0.09
AL	0.31**	0.48**	0.90**	0.35**	0.38**	0.42**	0.29**	0.28**	0.34**	0.22**	0.34**	0.24**	0.46**	− 0.25**	− 0.17**	− 0.34**	− 0.36**	0.19**
C	0.21**	0.22**	0.34**	0.24**	0.26**	0.32**	0.18**	0.04	0.17**	0.10	0.30**	0.21**	0.29**	− 0.09	− 0.20**	− 0.20**	− 0.19**	0.11
DI	0.22**	0.26**	0.35**	0.17**	0.19**	0.27**	0.13 *	0.17**	0.15 *	0.20**	0.24**	0.03	0.24**	− 0.02	− 0.12	− 0.15 *	− 0.14 *	0.11
FD	0.25**	0.37**	0.36**	0.28**	0.34**	0.34**	0.14 *	0.22**	0.38**	0.12	0.25**	0.19**	0.33**	− 0.18**	− 0.01	− 0.42**	− 0.11	0.05

*GS* Global scale; *PF* Physical functioning; *RF* Role functioning; *EF* Emotional functioning; *CF* Cognitive functioning; *SF* Social functioning; *F* Fatigue; *NV* Nausea and vomiting; *P* Pain; *DY* Dyspnea; *I* Insomnia; *AL* Appetite loss; *C* Constipation; *DI* Diarrhea; *FD* Financial difficulties; *P* Pain; *SW* Swallowing; *SN* Senses; *SP* Speech; *TSE* Trouble social eating;*TSC* Trouble social contact; *LS* Less sexuality; *T* Teeth; *OM* Opening mouth; *DM* Dry Mouth; *SS* Sticky saliva; *C* Coughing; *FI* Felt ill; *PK* Pain killers; *NS* Nutritional supplements; *FT* Feeding tube; *WL* Weight loss; *WG* Weight gain

*significant at *p* < 0.05;**significant at *p* < 0.01

The correlation of QoL (as measured by EORTC QLQ-H&N35) with resilience was assessed using Pearson correlation coefficients. Our results revealed a weak but significant correlation between 13 symptom domains of EORTC QLQ-H&N35 (Pain; Swallowing; Senses; Speech; Trouble Social Eating; Trouble Social Contact; Less Sexuality; Teeth; Opening Mouth; Dry Mouth; Sticky Saliva; Coughing; and Felt Ill) and resilience (r range: − 0.13 to − 0.50; *p* value < 0.001). However, a weak but significant positive correlation was observed between resilience and two domains (Nutritional Supplements and Weight Loss) and (r = 0.28 and 0.20, respectively; *p* value < 0.01).

When assessing the correlation between the EORCT QLQ-H&N 35 tool with depression and anxiety (HADS), we observed a significant weak-to-moderate positive correlation between 13 domains of symptoms scale of QLQ-H&N 35 (Pain; Swallowing; Senses; Speech; Trouble Social Eating; Trouble Social Contact; Less Sexuality; Teeth; Opening Mouth; Dry Mouth; Sticky Saliva; Coughing; and Felt Ill) with depression (r range: 0.19–0.42; *p* < 0.001) and with anxiety (r range: 0.26–0.67; *p* < 0.001). Additionally, there was a weak but significant negative correlation of 4 domains of symptom scale of QLQ-H&N 35 (Pain killers; Nutritional Supplements; Feeding tube; and Weight loss) with depression (r range: − 0.16 to 0.17; *p* < 0.001) and with anxiety (r range: − 0.17 to − 0.30; *p* < 0.001). Table 4 presents these results.

**Table 4:** Correlation between QLQ –H&N 35 with Resilience, Depression & Anxiety (Discriminant validity)

QLQ –H&N 35	Resilience score	Depression score	Anxiety score
*Symptoms*			
Pain	− 0.40 (< 0.001)*	0.40 (< 0.001)*	0.47 (< 0.001)*
Swallowing	− 0.27 (< 0.001)*	0.32 (< 0.001)*	0.47 (< 0.001)*
Senses problems	− 0.35 (< 0.001)*	0.42 (< 0.001)*	0.53 (0.001)*
Speech problems	− 0.40 (< 0.001)*	0.29 (< 0.001)*	0.50 (0.001)*
Trouble with social eating	− 0.454 (< 0.001)*	0.33 (< 0.001)*	0.50 (< 0.001)*
Trouble with social contact	− 0.50 (< 0.001)*	0.47 (< 0.001)*	0.67 (< 0.001)*
Less sexuality	− 0.40 (< 0.001)*	0.32 (< 0.001)*	0.39 (< 0.001)*
Teeth	− 0.13 (0.042)*	0.25 (< 0.001)*	0.31 (< 0.001)*
Opening mouth	− 0.18 (0.003)*	0.25 (< 0.001)*	0.35 (< 0.001)*
Dry mouth	− 0.13 (0.037)*	0.19 (0.002)*	0.26 (< 0.001)*
Sticky saliva	− 0.27 (< 0.001)*	0.22 (< 0.001)*	0.37 (< 0.001)*
Coughing	− 0.19 (0.003)*	0.15 (< 0.001)*	0.31 (< 0.001)*
Felt ill	− 0.44 (< 0.001)*	0.38 (< 0.001)*	0.52 (< 0.001)*
Pain killers	0.04 (0.503)	− 0.20 (< 0.001)*	− 0.17 (0.006)*
Nutritional supplements	0.28 (< 0.001)*	− 0.17 (0.002)*	− 0.24 (< 0.001)*
Feeding tube	0.07 (0.284)	− 0.17 (0.006)*	− 0.26 (< 0.001)*
Weight loss	0.20 (0.002)*	− 0.16 (0.012)*	− 0.30 (< 0.001)*
Weight gain	− 0.10 (0.131)	0.06 (0.327)	0.09 (0.180)

r = Pearson correlation coefficient

*significant at *p* value < 0.05

### Inter-scale correlations of EORTC QLQ-H&N35

When assessing inter-scale correlations of EORTC QLQ-H&N35, we observed a significant weak-to-strong positive correlation between 13 symptom domains of QLQ-H&N35 (Pain; Swallowing; Senses; Speech; Trouble Social Eating; Trouble Social Contact; Less Sexuality; Teeth; Opening Mouth; Dry Mouth; Sticky Saliva; and Coughing), with r ranging from 0.15 to 0.71 (*p* < 0.05). The strongest positive correlation was between Speech and Trouble in Social Eating (r = 0.71), Speech and Trouble in Social Contact (r = 0.64), Trouble in Social Eating and Trouble in Social Contact (r = 0.66), Feeling Ill and Trouble in Social Eating (r = 0.64), and between Feeling Ill and Trouble in Social Contact (r = 0.64). We also observed a significant weak-to-moderate negative correlation between 6 symptom domains of QLQ-H&N 35 (Felt ill; Pain Killers; Nutritional Supplements; Feeding tube; Weight Loss; and Weight Gain), with r ranging from − 0.14 to − 0.54 (*p* < 0.05). Table 5 presents these results.

**Table 5:** Interscale Correlation of EORTC QLQ-H&N35

	P	SW	SN	SP	TSE	TSC	LS	T	OM	DM	SS	C	FI	PK	NS	FT	WL	WG
P	1																	
SW	0.427**	1																
SN	0.376**	0.521**	1															
SP	0.302**	0.465**	0.398**	1														
TSE	0.411**	0.435**	0.447**	0.707**	1													
TSC	0.396**	0.450**	0.449**	0.642**	0.655**	1												
LS	0.167**	0.344**	0.347**	0.506**	0.531**	0.422**	1											
T	0.342**	0.325**	0.372**	0.320**	0.340**	0.328**	0.194**	1										
OM	0.361**	0.310**	0.353**	0.396**	0.497**	0.313**	0.246**	0.396**	1									
DM	0.178**	0.168**	0.280**	0.310**	0.394**	0.243**	0.245**	0.281**	0.346**	1								
SS	0.426**	0.388**	0.426**	0.379**	0.424**	0.335**	0.151*	0.360**	0.299**	0.352**	1							
C	0.238**	0.231**	0.257**	0.291**	0.183**	0.256**	0.002	0.144*	0.135*	0.0950	.252**	1						
FI	0.497**	0.575**	0.521**	0.622**	0.643**	0.621**	0.355**	0.341**	0.311**	0.297**	0.280**	0.309**	1					
PK	− 0.303**	− 0.146*	− 0.303**	− 0.062	− 0.192**	− 0.166**	− 0.036	− 0.272**	− 0.269**	− 0.105	− 0.148*	− − 0.088	− 0.287**	1				
NS	− 0.043	− 0.148*	− 0.188**	− 0.365**	− 0.362**	− 0.249**	− 0.439**	− 0.006	− 0.193**	− 0.123	− 0.078	− 0.070	− 0.222**	− 0.006	1			
FT	− 0.284**	− 0.418**	− 0.325**	− 0.405**	− 0.432**	− 0.299**	− 0.144*	− 0.251**	− 0.453**	− 0.131*	− 0.284**	− 0.301**	− 0.285**	0.143*	0.110	1		
WL	− 0.255**	− 0.328**	− 0.378**	− 0.338**	− 0.349**	− 0.307**	− 0.248**	− 0.146*	− 0.265**	− 0.109	− 0.257**	− 0.136*	− 0.330**	0.100	0.276**	0.307**	1	
WG	0.078	0.099	0.178**	0.055	0.118	0.096	0.057	− 0.054	0.090	− 0.018	0.028	0.028	0.134*	− 0.022	− 0.121	− 0.176**	− 0.541**	1

*P* Pain; *SW* Swallowing; *SN* Senses; *SP* Speech; *TSE* Trouble social eating; *TSC* Trouble social contact; *LS* Less sexuality; *T* Teeth; *OM* Opening mouth; *DM* Dry Mouth; *SS* Sticky saliva; *C* Coughing; *FI* Felt ill; *PK* Pain killers; *NS* Nutritional supplements; *FT* Feeding tube; *WL* Weight loss; *WG* Weight gain

*Significant at *p* < 0.05;**significant at *p* < 0.01

## Discussion

While health-related QoL is an important consideration in the management of patients with H&N cancers, the lack of suitable tools in Urdu precludes the assessment of QoL amongst patients in Pakistan. To this effect, we translated the EORTC QLQ-H&N35 into the Urdu language, and validated it in a sample of 250 patients with H&N cancer. The Cronbach alpha multi-item domains of the Urdu version of the QLQ-H&N35 ranged from 0.5 to 0.98, indicating variable internal consistency across domains. Moreover, high agreement was observed for both the expert-reported and patient-reported relevance (CVI: 0.88 and 0.92, respectively) and clarity (CVI: 0.84 and 0.93).

In our sample, the internal consistency of 6/7 multi-item symptom domains of QLQ-H&N35 ranged from acceptable to good (Cronbach’s alpha range: 0.75–0.98; *p* < 0.001), while the internal consistency of “Senses Problems” was subpar at 0.499. The low internal consistency of the “Senses Problems” multi-item scale was also seen in the validation of the Mexican [35], Hindi and Marathi [36], Cantonese [37], and Greek [38] versions of the QLQ-H&N35, as well when the QLQ-H&N35 was used in Norway, Sweden, the Netherlands [25], Arkansas [21], and Italy [39]. It has also been suggested that the items comprising “Senses Problems” be considered as individual items, rather than part of a multi-item scale [20]. Nevertheless, other translations, such as the Moroccan Arabic version (0.94) [24] and the Polish version (0.82) [40], demonstrate excellent internal consistency for “Senses Problems”.

Inter-scale correlations for the Urdu version of the EORTC QLQ-H&N35 revealed weak-to-strong positive correlation between 13 symptom domain of QLQ-H&N35 (Pain; Swallowing; Senses; Speech; Trouble Social eating; Trouble Social Contact; Less Sexuality; Teeth; Opening Mouth; Dry Mouth; Sticky Saliva; and Coughing). This pattern was also evident in the validation study by Sherman et al. conducted in Arkansas [21]. In addition, the Urdu version of the EORTC QLQ-H&N35 also demonstrated suitable discriminatory validity, evidenced by the distribution of floor and ceiling percentages.

The EORTC QLQ-H&N35 demonstrated good discriminant validity, with 13 symptom domains showing a weak negative correlation with resilience. Though conceptually it may be expected that resilience and QoL have a strong relationship, our results do little to affirm this hypothesis. Further work is required to explore the correlation of QoL and resilience amongst patients with H&N cancer. The EORTC QLQ-H&N35 also demonstrated a significant weak-to-moderate positive correlation between 13 symptom domains and depression and with anxiety. A similar study by Singer et al. also found only a weak-to-moderate correlation between the German translations of the EORTC QLQ-H&N35 and HADS [20]. The study by Sherman et al. demonstrated similar moderate correlation between the EORTC QLQ-H&N35 and depression and anxiety as measured by tools other than the HADS [21]. Thus, although our results show good discriminant validity of the EORTC QLQ-H&N35 with RS-14 and HADS, this may be due to mono-method bias. Mono-method bias arises when measurement of a construct is based of only a single tool for each construct (i.e., RS-14 for resilience and HADS for anxiety and depression), and it can pose a threat to discriminant validity by misrepresenting the true degree of association between constructs. Future studies may negate mono-method bias by measuring resilience, anxiety, and depression using multiple tools.

When assessing correlations between EORTC QLQ-30 and QLQ-H&N35, we observed a significant weak-to-moderate negative correlation of the 14 symptoms domains of QLQ-H&N35 with global QoL measured by the QLQ-30 and a significant weak-to-strong negative correlations with the functional domains of QLQ-30. These results are similar to those seen in the validation study by Sherman et al. [21], but unlike that of the Mexican translation [35] where excellent correlations were reported between EORTC QLQ-30 and QLQ-H&N35. Nevertheless, the weak correlations between the EORTC QLQ-30 and QLQ-H&N35 in our study indicate that although both measured QoL, they each assessed unique aspects of the construct. While the QLQ-30 focuses on overall QoL and functioning (physical, role, emotional, cognitive & social) with few generalized symptoms (fatigue, pain, nausea, and vomiting), the QLQ-H&N35 bases its QoL measurement predominantly off locoregional symptomatology (e.g., swallowing, speech problems, trouble with social eating, opening mouth, dry mouth, sticky saliva, coughing etc.). However, given the questionable convergent validity, it is important for clinicians and researchers to interpret QoL findings measured by the QLQ-H&N35 in the context of the QoL results provided by the more highly validated QLQ-C30.

The Urdu translation of the QLQ-H&N35 bears considerable importance for the management of H&N cancers in Pakistan. Considering social and cultural differences in Pakistan, and that most patients with H&N cancers belong to less educated backgrounds, it is crucial to have valid tools to assess QoL in a Pakistani setting. As Urdu is the national and official language of the country, it is understood and spoken throughout Pakistan. With the Urdu adaption of the QLQ-H&N35, the assessment of QoL may be incorporated as a routine feature in the management, prognostication, goal-setting, and monitoring of patients with H&N cancers in Pakistan. Moreover, it also provides a valid and reliable tool for clinical studies seeking to incorporate QoL measurement as part of their outcome assessment. Thus, this translation is a vital landmark for many stakeholders, including clinicians, patients, and cancer researchers.

For the most part, the translation and validation of the EORTC QLQ-H&N35 presented few challenges. The Urdu version was able to convey the intended English equivalents accurately, and was easily understandable to all the patients. This, coupled with the excellent internal consistency, and patient-reported clarity and relevance, confirm the Urdu version of the EORTC QLQ-H&N35 as a valid tool for the measurement of QoL amongst patients with H&N cancer in Pakistan. Moreover, our results also affirm the suitability of administration of the Urdu version of the EORTC QLQ-H&N35 via in-person interviews, which may be a necessity when the tool is used in populations with lower literacy rates. The setting of this study also adds to the generalizability and utility of the Urdu version of the EORTC QLQ-H&N35 across patient populations in Pakistan. The study took place in Karachi, the largest metropolitan city in Pakistan, and home to all major ethnicities in the country. Moreover, AKU, being one of the largest quaternary care hospitals in the surrounding regions, caters to diverse socioeconomic groups, as evidenced by the distribution of monthly family incomes.

However, our study has a few limitations. We did not perform test–retest analysis to investigate stability. Moreover, due to the cross-sectional nature of the study, we were unable to capture the temporal relationship between QoL, resilience, depression, and anxiety. Additionally, since our sample was recruited from a single center, our validation results may have limited generalizability to other centers in Pakistan. Lastly, our administration of the tool via patient interviews, as opposed to the recommended self-administered method, may have introduced interviewer or response biases. Future studies should aim to explore the interactions between QoL, resilience, and mental health longitudinally across extended periods of time, to better understand nuances in their relationship. Additionally, more sophisticated validation analyses of the Urdu versions of the EORTC QLQ-H&N35 must be performed to further judge its applicability in Pakistan.

## Conclusion

Our Urdu translation of the EORTC QLQ-H&N35 demonstrated validity comparable to translations in other languages validated in previous studies. The tool shows adequate discriminant validity when measured against resilience, depression, and anxiety. An issue of concern is the poor internal consistency of the “Senses Problems” multi-item domain. Further psychometric evaluation of the Urdu translation of the EORTC QLQ-H&N35 is necessary, to determine whether any changes are necessary to optimize validity in our population. With the Urdu adaption of the QLQ-H&N35, the assessment of QoL may be incorporated as a routine feature in the management, prognostication, goal-setting, and monitoring of patients with H&N cancers in Pakistan.

## Supplementary Information

**Additional file 1:** Final Minor Changes to Translation.

**Additional file 2:** Urdu Version of the EORTC QLQ-H&N35.

## Data Availability

The data that support the findings of this study are available from the corresponding author upon reasonable request.
