# Supplementary material for: Psychometric properties of the Urdu version of the EORTC QLQ-H&N35 (European organization for research and treatment of cancer head and neck module) quality of life tool
Source: BMC Psychol. 2022 Aug 7;10:194. doi: 10.1186/s40359-022-00900-x (PMC9358845; doi:10.1186/s40359-022-00900-x)
Supplement: Supplementary file 1 — Additional file 1: Final Minor Changes to Translation. [file 40359_2022_900_MOESM1_ESM.doc]

The difficulty that we faced was consensus of the two translators on a few terminologies in Urdu in some items of the tool. However, this problem was settled by a third independent translator after pretesting 10% of the participants.

For item 35 the phraseس طرح ا was replaced by کوئی مائع چیز. In item 36 the phrase شربے والی was replaced by گھٹی ہوئی. For item 38 the word شے was replaced by چیز and گھٹا دم to گلے میں پھنستی . For item 42, the word لعابwas replaced by تھوک. For items 43 and 44 the word تکلیف was replaced by دشواری. For item 46 the word کمزور was replaced by تبدیلی. For item 55 the phrase سماجی رابطے was replaced byمیل جول / میل ملاپ ا. For item 56 the phrase سماجی رابطے was replaced by ملنے میں. For item 58 the phrase میں مشکل سماجی رابطے was replaced by ملنے میں دشواری. For item 63 the phrase بزریعہ ٹیوب غزا فراہم was replaced by کو نالی کے ذریعے خوراک فراہم

**English Translation of the Above**

For item 35 the phrase “**things of that sort**” was replaced by "**any other liquids**". In item 36 the phrase “**curried foods**” was replaced by "**pureed foods**". For item 38 the word "**item**" was replaced by “**thing**” and “**suffocating**” to “**choking**”. For item 42, the word “**sputum**” was replaced by “**saliva**”. For items 43 and 44, the word “**discomfort**” was replaced by “**problem**”. For item 46 the word “**weak**” was replaced by “**changed**”. For item 55 the phrase “**societal communication**” to “**social interactions**”. For item 56 the phrase “**societal communication**” was replaced with “**social interactions**”. For item 58 the phrase “**difficulty in societal communication**” was replaced by “**problems in social contact**”. For item 63 the phrase “**tubal food administered**” was replaced by “**received food/meals via a tube**”.
