# Supplementary material for: Psychometric properties of the Urdu version of the EORTC QLQ-H&N35 (European organization for research and treatment of cancer head and neck module) quality of life tool
Source: BMC Psychol. 2022 Aug 7;10:194. doi: 10.1186/s40359-022-00900-x (PMC9358845; doi:10.1186/s40359-022-00900-x)
Supplement: Supplementary file 2 — Additional file 2: Urdu Version of the EORTC QLQ-H&N35. [file 40359_2022_900_MOESM2_ESM.doc]

## EORTC QLQ - H&N35 ( Head & Neck) (سر اور گردن)

|  | **During the past week** | **Response** | |
| --- | --- | --- | --- |
| 31. | Have you had pain in your mouth?  کیا آپ کو اپنے منہ میں درد محسوس ہوا؟ | 1. Not at All | بالکل بھی نہیں |
|  |  | 2. A little | تھوڑی سی |
|  |  | 3. Quite a Bit | کافی حد تک |
|  |  | 4.Very Much | بہت زیادہ |
| 32. | Have you had pain in your jaw?  کیا آپ کو اپنے جبڑے میں درد محسوس ہوا؟ | 1. Not at All | بالکل بھی نہیں |
|  |  | 2. A little | تھوڑی سی |
|  |  | 3. Quite a Bit | کافی حد تک |
|  |  | 4.Very Much | بہت زیادہ |
| 33. | Have you had soreness in your mouth?  کیا آپ کو اپنے منہ کو چھونے سے درد محسوس ہوا؟َ | 1. Not at All | بالکل بھی نہیں |
|  |  | 2. A little | تھوڑی سی |
|  |  | 3. Quite a Bit | کافی حد تک |
|  |  | 4.Very Much | بہت زیادہ |
| 34. | Have you had a painful throat?  کیا آپ کو گلے میں درد محسوس ہوا؟ | 1. Not at All | بالکل بھی نہیں |
|  |  | 2. A little | تھوڑی سی |
|  |  | 3. Quite a Bit | کافی حد تک |
|  |  | 4.Very Much | بہت زیادہ |

| 35. | Have you had problems swallowing liquids?  کیا آپ کو پانی /چائے یا کوئی مائع چیز نگلنے میں مشکل پیش آئی؟ | 1. Not at All | بالکل بھی نہیں |
| --- | --- | --- | --- |
|  |  | 2. A little | تھوڑی سی |
|  |  | 3. Quite a Bit | کافی حد تک |
|  |  | 4.Very Much | بہت زیادہ |
| 36. | Have you had problems swallowing pureed food?  کیا آپ کو گھٹی ہوئی غذا نگلنے میں دشواری پیش آئی؟ | 1. Not at All | بالکل بھی نہیں |
|  |  | 2. A little | تھوڑی سی |
|  |  | 3. Quite a Bit | کافی حد تک |
|  |  | 4.Very Much | بہت زیادہ |
| 37. | Have you had problems swallowing solid food?  کیا آپ کو ٹھوس غذا نگلنے میں مشکل پیش آئی؟ | 1. Not at All | بالکل بھی نہیں |
|  |  | 2. A little | تھوڑی سی |
|  |  | 3. Quite a Bit | کافی حد تک |
|  |  | 4.Very Much | بہت زیادہ |
| 38. | Have you choked when swallowing?  کیا کوئی چیزنگلتے وقت آپکے گلے میں پھنستی ہے؟ | 1. Not at All | بالکل بھی نہیں |
|  |  | 2. A little | تھوڑی سی |
|  |  | 3. Quite a Bit | کافی حد تک |
|  |  | 4.Very Much | بہت زیادہ |
| 39. | Have you had problems with your teeth?  کیا آپ کے دانتوں کے ساتھ کوئی مسئلہ ہواتھا؟َ | 1. Not at All | بالکل بھی نہیں |
|  |  | 2. A little | تھوڑی سی |
|  |  | 3. Quite a Bit | کافی حد تک |
|  |  | 4.Very Much | بہت زیادہ |
| 40. | Have you had problems opening your mouth wide?  کیا آپ کو اپنا منہ مکمل کھولنے میں مشکل پیش آئی؟ | 1. Not at All | بالکل بھی نہیں |
|  |  | 2. A little | تھوڑی سی |
|  |  | 3. Quite a Bit | کافی حد تک |
|  |  | 4.Very Much | بہت زیادہ |

| 41. | Have you had a dry mouth?  کیا آپ کو اپنا منہ خشک لگا؟ | 1. Not at All | بالکل بھی نہیں |
| --- | --- | --- | --- |
|  |  | 2. A little | تھوڑی سی |
|  |  | 3. Quite a Bit | کافی حد تک |
|  |  | 4.Very Much | بہت زیادہ |
| 42. | Have you had sticky saliva?  کیا آپ کے منہ کا تھوک چپکنےوالا تھا؟ | 1. Not at All | بالکل بھی نہیں |
|  |  | 2. A little | تھوڑی سی |
|  |  | 3. Quite a Bit | کافی حد تک |
|  |  | 4.Very Much | بہت زیادہ |
| 43. | Have you had problems with your sense of smell?  کیا آپ کو اپنے سونگھنے کی حس میں کوئی دشواری محسوسں ہوئی؟ | 1. Not at All | بالکل بھی نہیں |
|  |  | 2. A little | تھوڑی سی |
|  |  | 3. Quite a Bit | کافی حد تک |
|  |  | 4.Very Much | بہت زیادہ |
| 44. | Have you had problems with your sense of taste?  کیا آپ کو ذائقے کی حس میں کوئی دشواری ہوئی؟ | 1. Not at All | بالکل بھی نہیں |
|  |  | 2. A little | تھوڑی سی |
|  |  | 3. Quite a Bit | کافی حد تک |
|  |  | 4.Very Much | بہت زیادہ |
| 45. | Have you coughed?  کیا آپ کو کھانسی ہوئی ہے؟ | 1. Not at All | بالکل بھی نہیں |
|  |  | 2. A little | تھوڑی سی |
|  |  | 3. Quite a Bit | کافی حد تک |
|  |  | 4.Very Much | بہت زیادہ |
| 46. | Have you been hoarse Or have change of voice / articulation/ pronunciation?)  کیا آپ کی آواز میں تبدیلی ہوئ تھی ؟ | 1. Not at All | بالکل بھی نہیں |
|  |  | 2. A little | تھوڑی سی |
|  |  | 3. Quite a Bit | کافی حد تک |
|  |  | 4.Very Much | بہت زیادہ |

| 47. | Have you felt ill?  کیا آپ نے خود کو بیمار محسوس کیا؟ | 1. Not at All | بالکل بھی نہیں |
| --- | --- | --- | --- |
|  |  | 2. A little | تھوڑی سی |
|  |  | 3. Quite a Bit | کافی حد تک |
|  |  | 4.Very Much | بہت زیادہ |
| 48. | Has your appearance bothered you?  کیا آپ کے حلئے نے آپ کو پریشان کیا؟َ | 1. Not at All | بالکل بھی نہیں |
|  |  | 2. A little | تھوڑی سی |
|  |  | 3. Quite a Bit | کافی حد تک |
|  |  | 4.Very Much | بہت زیادہ |
| 49. | Have you had trouble eating?  کیا آپ کو کھانے میں دشواری ہوئی ؟ | 1. Not at All | بالکل بھی نہیں |
|  |  | 2. A little | تھوڑی سی |
|  |  | 3. Quite a Bit | کافی حد تک |
|  |  | 4.Very Much | بہت زیادہ |
| 50. | Have you had trouble eating in front of your family?  کیا آپ کو اپنے خاندان کے افراد کے سامنے کھانے میں دشواری ہوئی؟ | 1. Not at All | بالکل بھی نہیں |
|  |  | 2. A little | تھوڑی سی |
|  |  | 3. Quite a Bit | کافی حد تک |
|  |  | 4.Very Much | بہت زیادہ |
| 51. | Have you had trouble eating in front of other people?  کیا آپ کو دیگر لوگوں کے سامنے کھانے میں دشواری ہوئی؟ | 1. Not at All | بالکل بھی نہیں |
|  |  | 2. A little | تھوڑی سی |
|  |  | 3. Quite a Bit | کافی حد تک |
|  |  | 4.Very Much | بہت زیادہ |
| 52. | Have you had trouble enjoying your meals?  کیا آپ کو اپنے کھانوں سے لطف اٹھانے میں دشواری ہوئی؟ | 1. Not at All | بالکل بھی نہیں |
|  |  | 2. A little | تھوڑی سی |
|  |  | 3. Quite a Bit | کافی حد تک |
|  |  | 4.Very Much | بہت زیادہ |

| 53. | Have you had trouble talking to other people?  کیا آپ کو دوسرے افراد سے بات کرنے میں دشواری ہوئی ؟ | 1. Not at All | بالکل بھی نہیں |
| --- | --- | --- | --- |
|  |  | 2. A little | تھوڑی سی |
|  |  | 3. Quite a Bit | کافی حد تک |
|  |  | 4.Very Much | بہت زیادہ |
| 54. | Have you had trouble talking on the telephone  (***Due to difficulty in hearing/due to surgery, scar, fibrosis due to radiation)***?  کیا آپ کو فون پر بات کرنے میں دشواری ہوئی ؟ | 1. Not at All | بالکل بھی نہیں |
|  |  | 2. A little | تھوڑی سی |
|  |  | 3. Quite a Bit | کافی حد تک |
|  |  | 4.Very Much | بہت زیادہ |
| 55. | Have you had trouble having social contact with your family?  کیا آپ کو اپنے اہل خانہ سے میل جول / میل ملاپ میں دشواری ہوئی ؟ | 1. Not at All | بالکل بھی نہیں |
|  |  | 2. A little | تھوڑی سی |
|  |  | 3. Quite a Bit | کافی حد تک |
|  |  | 4.Very Much | بہت زیادہ |
| 56. | Have you had trouble having social contact with friends?  کیا آپ کو اپنے دوستوں سے ملنے میں دشواری ہوئی؟ | 1. Not at All | بالکل بھی نہیں |
|  |  | 2. A little | تھوڑی سی |
|  |  | 3. Quite a Bit | کافی حد تک |
|  |  | 4.Very Much | بہت زیادہ |
| 57. | Have you had trouble going out in public?  کیا آپ کو گھرسے باہرعام لوگوں میں جانے میں دشواری ہوئی؟ | 1. Not at All | بالکل بھی نہیں |
|  |  | 2. A little | تھوڑی سی |
|  |  | 3. Quite a Bit | کافی حد تک |
|  |  | 4.Very Much | بہت زیادہ |
| 58. | Have you had trouble having physical contact with family or friends?  کیا آپ کو اہل خانہ یا دوستوں کے ساتھ ملنےمیں دشواری پیش آئی؟ | 1. Not at All | بالکل بھی نہیں |
|  |  | 2. A little | تھوڑی سی |
|  |  | 3. Quite a Bit | کافی حد تک |
|  |  | 4.Very Much | بہت زیادہ |

| 59. | Have you felt less interest in sex?  کیا آپ کو جنسی رابطہ/ تعلق میں کم دلچسپی محسوس ہوئی؟ | 1. Not at All | بالکل بھی نہیں |
| --- | --- | --- | --- |
|  |  | 2. A little | تھوڑی سی |
|  |  | 3. Quite a Bit | کافی حد تک |
|  |  | 4.Very Much | بہت زیادہ |
| 60. | Have you felt less sexual enjoyment?  کیا آپ نے جنسی لطف کم محسوس کیا؟ | 1. Not at All | بالکل بھی نہیں |
|  |  | 2. A little | تھوڑی سی |
|  |  | 3. Quite a Bit | کافی حد تک |
|  |  | 4.Very Much | بہت زیادہ |
| 61. | Have you used pain-killers?  کیا آپ نے درد دور کرنے والی ادویات لیں؟ | 1. Yes | جی ہاں |
|  |  | 1. No | جی نہیں |
| 62. | Have you taken any nutritional supplements (excluding vitamins)?  کیا آپ نے کوئی اضافی غذائیت بخش خوراک لیں (وٹامنس کے علاوہ)؟ | 1. Yes | جی ہاں |
|  |  | 1. No | جی نہیں |
| 63. | Have you used a feeding tube?  کیا آپ کو نالی کے ذریعے خوراک فراہم کی گئی؟ | 1. Yes | جی ہاں |
|  |  | 1. No | جی نہیں |
| 64. | Have you lost weight?  کیا آپ کا وزن کم ہوا؟ | 1. Yes | جی ہاں |
|  |  | 1. No | جی نہیں |
| 65. | Have you gained weight?  کیا آپ کا وزن بڑھاہے؟ | 1. Yes | جی ہاں |
|  |  | 1. No | جی نہیں |
